# A Rare Cause of Female Virilization: A Case Report

**DOI:** 10.7759/cureus.96744

**Published:** 2025-11-13

**Authors:** Rodrigo Realista, Beatriz Neves, Julia Pereira, Ana Rita Pinto

**Affiliations:** 1 Obstetrics and Gynecology, Centro Hospitalar Universitário de São João, Porto, PRT; 2 Obstetrics and Gynecology, Hospital Pedro Hispano, Matosinhos, PRT; 3 Gynecologic Oncology, Unidade Local de Saúde da Póvoa de Varzim e Vila do Conde, Porto, PRT; 4 Gynecologic Oncology, Instituto Português de Oncologia do Porto Francisco Gentil, EPE, Porto, PRT

**Keywords:** dicer 1 mutation, female hyperandrogenism, ovarian cancer surgery, sertoli-leydig cell tumour, virilization

## Abstract

Sertoli-Leydig cell tumors (SLCTs) are uncommon ovarian sex cord stromal neoplasms that typically affect young women and may cause a rise in androgen production. We report a case of a 21-year-old woman who sought medical care because of a two-year amenorrhea, progressive virilization, and a large abdominopelvic mass. Imaging revealed a solid multiloculated adnexal lesion measuring 200 x 80 x 150 mm, associated with a marked hyperandrogenism (total testosterone 584 ng/dL). She underwent surgery for a left adnexectomy, in which no extraovarian involvement was noticed. Histopathology disclosed a moderately differentiated SLCT with mixed Sertoli and Leydig elements, high mitotic activity, and positivity for WT1, CAM5.2, inhibin A, vimentin, and calretinin. Peritoneal cytology was negative, which classified the disease stage as IA. Tumor sequencing identified two somatic DICER1 variants, although not present in peripheral blood, thus excluding a DICER1 syndrome.

The patient recovered uneventfully, and at six months after surgery, androgen levels and clinical virilization signs had resolved. Given their usual early presentation, SLCTs are primarily managed surgically with conservative unilateral resection. Prognosis correlates with differentiation and stage. Periodically, clinical and biochemical surveillance is advised, and molecular testing can guide genetic counseling and further management.

## Introduction

Sertoli-Leydig cell tumors (SLCTs), also called androblastoma, are uncommon ovarian tumors originating from the sex cord stromal cells, and accounting for <0.2% of all ovarian malignancies [1]. Despite being found in women of all ages, they are more common in women in the third decade of life [2]. Although they are the most frequent cause of virilizing ovarian tumors, they are a rare cause of hirsutism. Since they are diagnosed at a young age, fertility-sparing surgery is usually preferred. The majority of patients are diagnosed at an early stage of the disease.

In this article, we intend to report the case of a female patient in their 20s who sought medical care for a two-year amenorrhea with virilization signs and an abdominal mass, found to be a SLCT. We aim to review the literature concerning the diagnosis, staging, and treatment of these tumors. We ensure that the work has been reported in line with the Surgical CAse REport (SCARE) 2023 criteria.

## Case presentation

**Figure 1:**
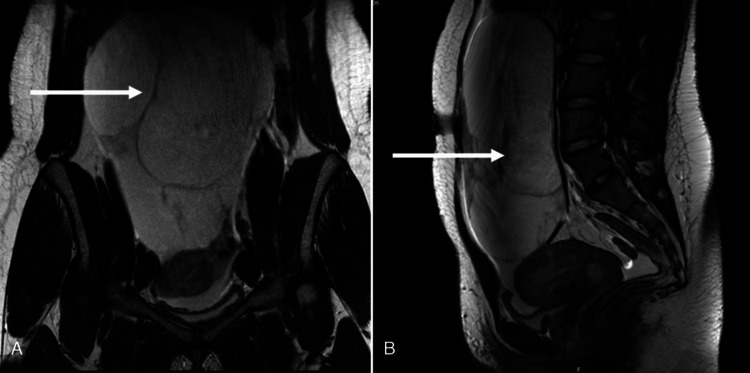
(A-B) Coronal and sagittal MRI images of the pelvic mass (arrows)

In this article, we explore the case of a 21-year-old female patient, having no underlying medical disease, who was referred by her family physician for an amenorrhea for the previous two years, with progressive abdominal distention and excessive hair growth on her limbs, face, and chest for the previous eight months. On physical examination, she exhibited extensive hirsutism, clitoromegaly, and a distended abdomen, with a mass occupying the totality of the abdominal cavity. At admission, her vital signs were normal, and gynecologic examination showed an extensive mass obliterating the anterior cul-de-sac. The pelvic ultrasound showed a normal uterus, with a thin endometrial lining, and a pelvic solid mass whose limits were impossible to define by transvaginal ultrasound. This appeared to be a multiseptated solid mass with regular walls, showing no cystic component or vascular invasion by Doppler, located in the left adnexal area. By abdominal ultrasound, the mass occupied the totality of the abdominal cavity until the epigastric area, being classified as an O-RADS4. The MRI showed a pelvic mass with 200 x 80 x 150 mm, originating from the left adnexal area, characterized by multiple septae, showing no abdominal lymphadenopathy or abdominal deposits (Figure 1).

The hormonal assessment showed excessive androgenic activity in the form of an elevated total serum testosterone level of 584 ng/dL (8.4-48.1 ng/dL) and free testosterone of 1.82 ng/dL (0.07-0.38 ng/dL). The level of alpha-fetoprotein (AFP) was also elevated 17 ng/mL (<7 ng/mL) and the levels of the remaining ovarian markers - cancer antigen 125 (CA 125), cancer antigen 19-9 (CA 19-9), cancer antigen 15-3 (CA 15-3), carcinoembryonic antigen (CEA), lactate dehydrogenase (LDH), human chorionic gonadotropin (hCG) and inhibin-α - were normal (Table 1).

**Table 1:** Laboratory tests with reference range CA 125: cancer antigen 125; CA 19-9: cancer antigen 19-9; CA 15-3: cancer antigen 15-3; CEA: carcinoembryonic antigen; LDH: lactate dehydrogenase; hCG: human chorionic gonadotropin

Marker	Value	Normal range
Total testosterone	584 ng/dL	8.4-48.1 ng/dL
Free testosterone	1.82 ng/dL	0.07-0.38 ng/dL
Alpha-fetoprotein	17 ng/mL	<7 ng/mL
CA 125	≤12 kU/mL	≤35 kU/mL
CA 19-9	9 U/mL	≤37 U/mL
CA 15-3	15.3 U/mL	≤30 U/mL
CEA	1.7 µg/L	≤3.0 µg/L
LDH	156 U/L	140 to 280 U/L
hCG	1.3 mU/mL	<5 mU/mL
Inhibin-α	10 pg/mL	5-160 pg/mL

**Figure 2:**
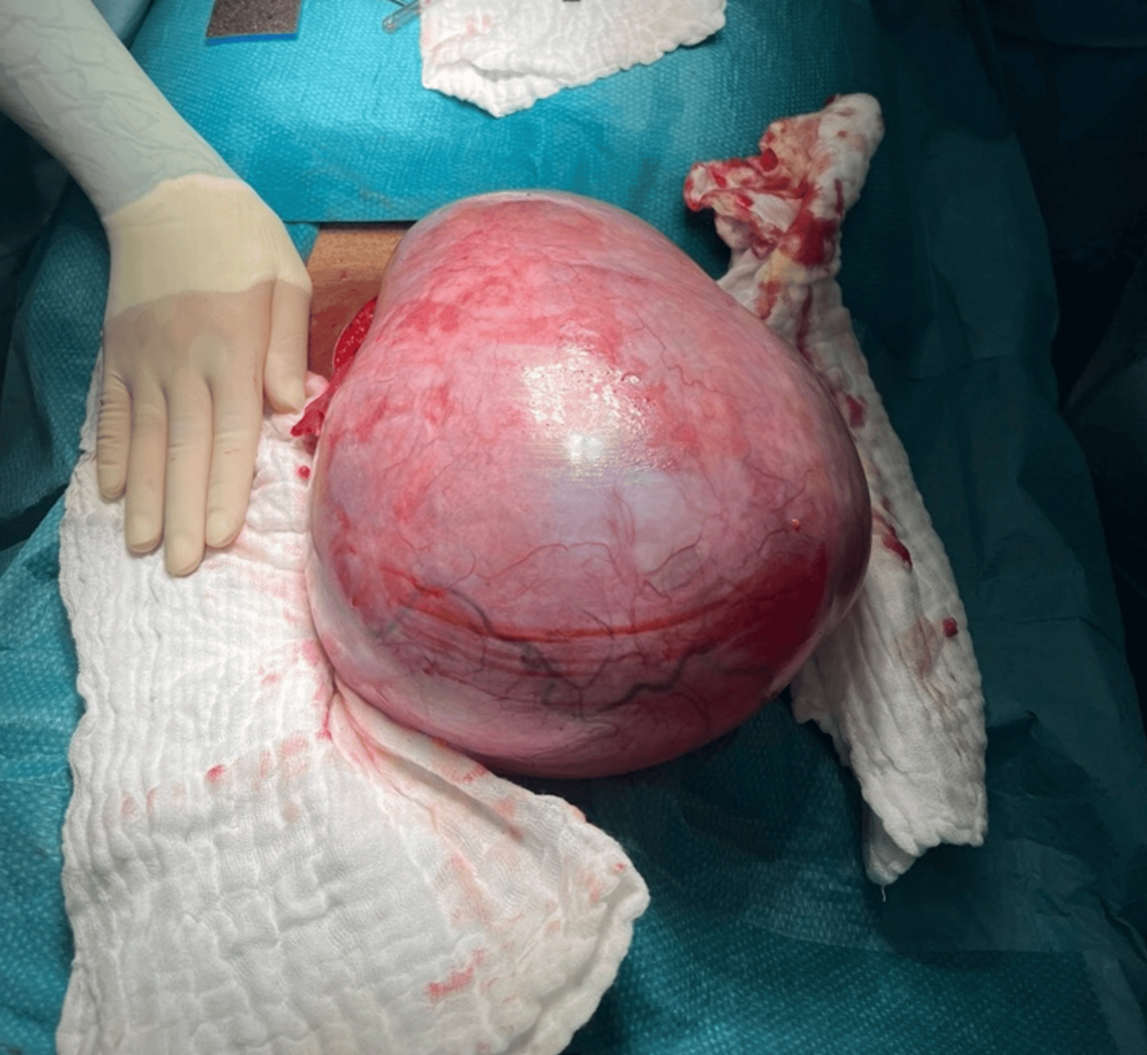
Intraoperative view of the ovarian mass during laparotomic exploration

Concerning these findings, a presumptive diagnosis of an androgen-secreting ovarian tumor was made. A laparotomic surgical exploration was performed, with the visualization of a 250 mm ovarian mass, with a smooth surface (Figure 2).

The right ovary and uterus were normal; there were no pelvic adhesions, the abdominal cavity was regular, and no signs of carcinosis. The appendix, the liver, spleen, and kidneys were normal, with no peritoneal nodules. Peritoneal cytology was performed, followed by a left adnexectomy. The post-surgery period had no adverse events, and the patient recovered to was discharged three days after the surgery. Macroscopically, the left adnexa weighs 3611 g, with a normal fallopian tube and an ovary measuring 240 x 220 x 90 mm, with a smooth surface and multiseptated walls, with a serous content. Microscopically, the pathology report showed a neoplasia with variable cell density, with areas of dense cellularity and others of loose stroma. The architecture was heterogeneous with solid, trabecular, cord-like, cystic, nest-like, tubular, and follicular areas. The cells were predominantly small, with round, uniform nuclei and scant cytoplasm. Cells with more abundant eosinophilic cytoplasm (Leydig cells) were also identified, arranged in multiple aggregates dispersed throughout the tumor. The mitotic index was 28 mitoses per 10 high-power fields. It showed no lymphovascular invasion. The tumor cells stained positive for Wilms tumor protein 1 (WT1), cytokeratin CAM5.2, inhibin-α, vimentin, and calretinin. The peritoneal fluid analysis showed no abnormal cells. Based on clinical, histopathological, and immunohistochemical findings, a diagnosis of moderately differentiated stage IA STLC was made.

Concerning the genetic analysis, two DICER1 variants were found in tumor cells (one missense mutation that is a characteristic somatic hotspot and a nonsense mutation not previously described), raising concern of a possible germ-line mutation related to this gene. However, the peripheral blood analysis didn’t show any of these variants.

She was observed six months after surgery, showing no signs of hyperandrogenism, and analytical improvement: total serum testosterone level of 32 ng/dL (8.4-48.1 ng/dL) and free testosterone of 0.12 ng/dL (0.07-0.38 ng/dL).

## Discussion

Despite being a rare type of ovarian malignancy, SLCT should be suspected in the presence of hyperandrogenism signs and pelvic mass, since they are the most frequent cause of virilizing ovarian tumors [2,3]. These hyperandrogenism signs can be found in 40% of the cases, and consist of hirsutism, menstrual disorders such as oligo-menorrhea or amenorrhea, clitoral and labia majora hypertrophy, and hoarseness of the voice.

In the presented case, the patient sought medical care for amenorrhea and virilization signs, which reflected the increased testosterone levels. These virilization signs are usually seen with blood testosterone levels above 200 ng/mL [4], values that largely surpass the physiological range of female testosterone levels of 10-55 ng/mL. These tumors are almost exclusively unilateral, with an average size of 12-14 cm [5]. Depending on the histological type, they are usually limited to the ovary, with only 2% presenting with metastasis at diagnosis (mainly poorly-differentiated) [5].

Imaging features are nonspecific and variable [4,6], making it difficult to make a diagnosis based on radiologic findings. In ultrasound, SLCTs typically present as a solid mass with intratumoral anechoic fluid areas at ultrasound, with a multiloculated appearance.

MRI scans are reserved for tumor characterization and extension assessment for surgical purposes, showing predominantly low T2 signal intensity with scattered areas of high signal. It is also important for the assessment of lymphatic invasion, although this is a rare finding [6,7].

Macroscopically, SLCTs exhibit a solid, fleshy, yellow, and lobulated cut surface with focal cyst formation. Histologically, they are classified into five subtypes: (1) well-differentiated; (2) moderately-differentiated; (3) poorly-differentiated; (4) retiform; and (5) those with heterologous elements. The main finding that allows this classification is the degree of Sertoli tubular differentiation, the proportion of Leydig cell component, and the quantity of primitive gonadal stroma. Well-differentiated are the least common subtype, and contain sertoliform tubes with interspersed Leydig cells. In moderately-differentiated Sertoli components show a mix of solid, corded, and tubular growth patterns, while poorly-differentiated show solid growth with sarcomatoid features. Retiform SLCTs have anastomosed slit-like openings, surrounded by columnar epithelium [5]. Prognosis is inversely related to the degree of differentiation. Immunohistochemistry markers typical of sex cord stroma are positive: inhibin-α, WT-1, calretinin, and vimentin. Nevertheless, these markers don’t allow the differentiation between SLCT and other sex cord stromal cell tumors.

The patient's age, tumor stage, and degree of tumor differentiation are the most important prognostic factors for these patients. A formal surgical staging approach should be performed in all patients, particularly with intermediate and high-grade tumors. Treatment options are often difficult to choose because, due to the rarity of the disease, there are no standard treatment regimens defined for SLCTs, especially for adjunct therapy.

Since well-differentiated tumors are always considered benign, and most of the time confined to one ovary, fertility-sparing approaches, such as unilateral adnexectomy, are usually preferred in young patients [8-10]. In cases where there is no wish to preserve fertility, a bilateral adnexectomy with hysterectomy should be considered. In low-risk disease (stages IA and IB), a surveillance approach can be carried out after surgery. Adjuvant therapy should be considered in high-risk disease, such as stage IC or poorly-, moderately-differentiated, retiform, those with heterologous elements, and stages II-IV. Bleomycin, etoposide, and cisplatin (BEP) appear to be an active combination regimen for first-line chemotherapy [8-10]. Other chemotherapy regimens used in the literature are carboplatin and paclitaxel [10]. Radiotherapy is not indicated for adjuvant therapy or after R0 resection, since there is a lack of evidence for these tumor radiosensitivities.

Prognosis depends largely on the degree of tumor differentiation: five-year survival of 100% for well-differentiated tumors; 80% for moderately to poorly differentiated forms. The five-year survival drops to near 0% for metastatic disease [8-10]. Although these tumors generally have a good prognosis, relapse rates up to 33% have been described [11], with most of them occurring in the first two years of disease, and being rare after five years [8].

Surveillance recommendations are not well defined. However, a clinical and analytic monitoring should be performed (if there was an androgen elevation prior to therapy) every four months during the first two years, every six months between the third and the fight year, and annually beyond the sixth follow-up year, until the rest of their lives. Pelvic ultrasound can be performed for monitoring of the contralateral ovary, and there is no evidence for routine imagiologic monitoring, unless a relapse is suspected [8]. The prognosis for relapse is poor, with a salvage rate <20% for clinically malignant and recurrent disease [12,13]. The literature also reports a very poor prognosis for relapse, with a salvage rate of <20% for clinically malignant and recurrent disease [12,13].

Tumor cells' genetic analysis is crucial to rule out the minority of cases that are associated with genetic syndromes. Two DICER1 variants were found in tumor cells. DICER1 syndrome is a rare genetic disorder caused by germline loss-of-function variants.

It is an autosomal dominant disorder that predisposes to a variety of tumors (both malignant and benign). In this particular case, if the mutation was found in peripheral blood, the clinical management would be different since the patient should be submitted to bilateral adnexectomy, and start a surveillance program [14]. Since the peripheral blood analysis didn’t show any of the variants found in tumor cells, and the patient doesn’t have other clinical characteristics (namely pleuropulmonary blastoma or pulmonary cysts), DICER1 syndrome was ruled out.

## Conclusions

SLCT can be challenging to diagnose. Since they present at early stages in the majority of cases, their treatment is essentially surgical. Prognosis is good, with a low relapse rate. Due to the rarity of these tumors, optimal adjuvant chemotherapy regimens remain to be defined. Clinicians should be aware of the clinical presentation of these tumors to intervene in early stages, seeking a fertility-sparing approach whenever possible. Efforts should be made to notify these cases in order to create international registries for a better approach to high-risk cases.
